# Association between comorbidity burden and outcomes of catheter ablation vs. medical therapy for atrial fibrillation: insights from the CABANA trial

**DOI:** 10.1093/europace/euaf292

**Published:** 2025-11-10

**Authors:** Yang Chen, Eva Soler-Espejo, Manlin Zhao, Wenhui Li, Hongyu Liu, Ying Gue, Garry McDowell, Douglas L Packer, Gregory Y H Lip

**Affiliations:** Liverpool Centre for Cardiovascular Science at University of Liverpool, Liverpool John Moores University and Liverpool Heart & Chest Hospital, William Henry Duncan Building, 6 West Derby Street, Liverpool L7 8TX, UK; Department of Cardiovascular and Metabolic Medicine, Institute of Life Course and Medical Sciences, University of Liverpool, Liverpool, UK; Liverpool Centre for Cardiovascular Science at University of Liverpool, Liverpool John Moores University and Liverpool Heart & Chest Hospital, William Henry Duncan Building, 6 West Derby Street, Liverpool L7 8TX, UK; Department of Hematology, Hospital Clínico Universitario Virgen de la Arrixaca, University of Murcia, Instituto Murciano de Investigación Biosanitaria (IMIB-Arrixaca), Murcia, Spain; Liverpool Centre for Cardiovascular Science at University of Liverpool, Liverpool John Moores University and Liverpool Heart & Chest Hospital, William Henry Duncan Building, 6 West Derby Street, Liverpool L7 8TX, UK; Department of Cardiology, Beijing Anzhen Hospital, Capital Medical University, Engineering Research Center of Medical Devices for Cardiovascular Diseases, Ministry of Education, National Clinical Research Center for Cardiovascular Diseases, Beijing, People’s Republic of China; Liverpool Centre for Cardiovascular Science at University of Liverpool, Liverpool John Moores University and Liverpool Heart & Chest Hospital, William Henry Duncan Building, 6 West Derby Street, Liverpool L7 8TX, UK; Department of Computer Science, Faculty of Environment, Science and Economy, University of Exeter, Exeter, UK; Liverpool Centre for Cardiovascular Science at University of Liverpool, Liverpool John Moores University and Liverpool Heart & Chest Hospital, William Henry Duncan Building, 6 West Derby Street, Liverpool L7 8TX, UK; Department of Medical Genetics, the Second Affiliated Hospital, Jiangxi Medical College, Nanchang University, Nanchang, Jiangxi, People’s Republic of China; Liverpool Centre for Cardiovascular Science at University of Liverpool, Liverpool John Moores University and Liverpool Heart & Chest Hospital, William Henry Duncan Building, 6 West Derby Street, Liverpool L7 8TX, UK; Liverpool Centre for Cardiovascular Science at University of Liverpool, Liverpool John Moores University and Liverpool Heart & Chest Hospital, William Henry Duncan Building, 6 West Derby Street, Liverpool L7 8TX, UK; School of Pharmacy and Biomolecular Sciences, Liverpool John Moores University, Liverpool, UK; Department of Cardiology, Intermountain Medical Center, Murray, UT, USA; Liverpool Centre for Cardiovascular Science at University of Liverpool, Liverpool John Moores University and Liverpool Heart & Chest Hospital, William Henry Duncan Building, 6 West Derby Street, Liverpool L7 8TX, UK; Department of Clinical Medicine, Aalborg University, Fredrik Bajers Vej 7, Aalborg 9220, Denmark; Department of Cardiology, Lipidology and Internal Medicine, Medical University of Bialystok, ul. Żurawia 14, Bialystok 15-540, Poland

**Keywords:** Atrial fibrillation, CABANA trial, Catheter ablation, Comorbidity burden, Multimorbidity

## Abstract

**Aims:**

Multimorbidity frequently coexists with atrial fibrillation (AF) and complicates treatment decisions. While current guidelines offer selective recommendations for catheter ablation in this group, evidence remains limited. This study aimed to evaluate whether comorbidity burden modifies the effectiveness of catheter ablation vs. antiarrhythmic drug therapy.

**Methods and results:**

In this *post hoc* analysis of the CABANA trial, patients were stratified by overall comorbidity burden using a data-driven threshold based on the distribution of 15 pre-specified conditions. The *primary outcome* was a composite of all-cause mortality, disabling stroke, serious bleeding, or cardiac arrest. *Secondary outcomes* included cardiovascular hospitalization and a composite of all-cause mortality or cardiovascular hospitalization. *Additional outcomes* included AF recurrence and AF-related quality of life in a sub-cohort. Of 2204 patients, 736 had high comorbidity burden {≥4 conditions, based on a data-driven threshold; median age 68.0 [interquartile range (IQR): 63.0–73.0], 67.1% male} and 1468 had low burden [median age 67.0 (IQR: 61.0–71.0), 60.7% male]. Over a median follow-up of 3.9 years (IQR: 2.4–5.1), for the primary outcome, the adjusted hazard ratio for catheter ablation vs. drug therapy was 0.62 [95% confidence interval (CI): 0.42–0.93] in patients with high comorbidity burden and 1.16 (95% CI: 0.76–1.77) in those with low burden (interaction *P* = 0.038). Secondary outcomes also tended to favour ablation in the high comorbidity burden group. Moreover, catheter ablation significantly reduced AF recurrence, with relative risk reductions of 49% and 40% in the low- and high-burden groups, respectively. Furthermore, catheter ablation improved AF-related quality of life in both comorbidity groups, with more sustained and pronounced benefits over time in patients with high comorbidity burden.

**Conclusion:**

Catheter ablation was associated with more favourable clinical outcomes in AF patients with high comorbidity burden, which support broader consideration of ablation in this population, though prospective trials are needed to confirm and guide clinical decision-making in personalized rhythm management.

**Pre-registered clinical trial number:**

NCT00911508

## Graphical Abstract

**Graphical Abstract:**
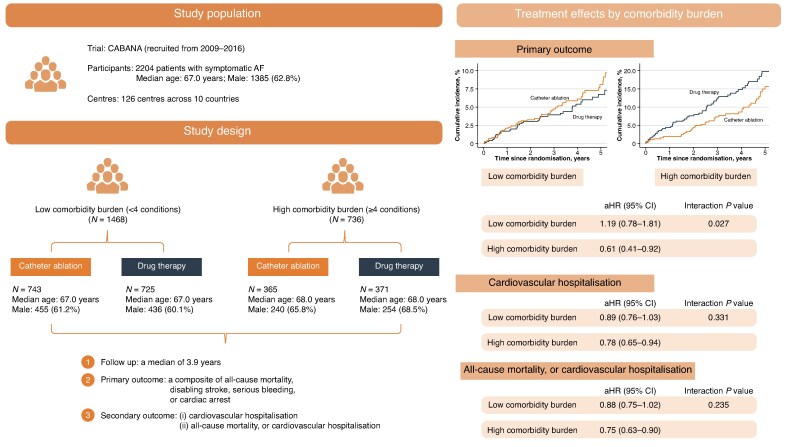

What’s new?

- This is a *post hoc* analysis of the CABANA randomized trial evaluating whether comorbidity burden modifies the long-term effectiveness of catheter ablation vs. drug therapy in patients with atrial fibrillation (AF) (*n* = 2 204).
- Over a median follow-up of 3.9 years, catheter ablation was associated with more favourable clinical outcomes in patients with a high comorbidity burden, while no such benefit was observed in those with lower burden, suggesting treatment effect modification by comorbidity profile.
- Patients with a high comorbidity burden experienced more sustained and pronounced improvements in AF-related quality of life following catheter ablation, highlighting its potential to deliver durable symptomatic relief in complex patient populations.

## Introduction

With global population ageing and the rising burden of chronic diseases, atrial fibrillation (AF) is increasingly recognized as a condition commonly characterized by complex multimorbidity.^1^ The presence of multimorbidity in AF patients contributes to more severe symptoms, advanced AF progression, higher mortality risk, and heightened cardiovascular risk.^2–4^

Nevertheless, multimorbidity, which is often defined as two or more chronic conditions, lacks a standardized definition. Real-world studies have reported a high comorbidity burden among AF patients, with one finding that over half had three to five comorbidities^5^ and another reporting that ≥4 were present in more than 75% of patients.^6^ These reinforce the need to incorporate multimorbidity assessment into AF care, as endorsed by current guidelines.^7,8^

Catheter ablation is an effective rhythm control strategy in AF, mainly by reducing recurrence and hospitalization rates.^7–9^ The Catheter Ablation vs. Antiarrhythmic Drug Therapy for Atrial Fibrillation (CABANA) trial, the largest randomized controlled trial (RCT) comparing catheter ablation with drug therapy, reported no significant reduction in adverse outcomes with ablation compared to antiarrhythmic drug therapy.^10^ Consequently, there is increasing interest in identifying AF patient profiles that may benefit preferentially from catheter ablation. Given that AF patients with multimorbidity often experience greater frailty, polypharmacy, and organ dysfunction,^11,12^ the risk–benefit profile of rhythm control strategies may differ substantially in this group.

Current guidelines acknowledge this heterogeneity to varying degrees. The 2023 American Heart Association/American College of Cardiology/Heart Rhythm Society (AHA/ACC/HRS) guidelines recommend catheter ablation as a first-line treatment (Class I) for younger, relatively healthy patients with symptomatic paroxysmal AF and as a Class IIa option for older individuals with multiple comorbidities.^8^ In contrast, the 2024 European Society of Cardiology/European Heart Rhythm Association/European Association for Cardio-Thoracic Surgery (ESC/EHRA/EACTS) guidelines provide a Class I recommendation for catheter ablation as a first-line strategy in patients with paroxysmal AF,^7,13^ without stratifying by comorbidity status. However, these recommendations are largely derived from trials conducted in lower-risk populations,^14–18^ and it remains uncertain whether similar benefits extend to those with high comorbidity burden. Whether multimorbidity attenuates or amplifies the therapeutic benefit of catheter ablation remains a critical evidence gap with important implications for personalized care.

Therefore, this CABANA sub-analysis aimed to evaluate whether the overall burden of diverse comorbidities modifies the long-term effectiveness of catheter ablation vs. drug therapy in AF.

## Methods

### Trial design, intervention, and study population

Details regarding the design, rationale, and primary outcomes of the CABANA trial (URL: https://clinicaltrials.gov; unique identifier: NCT00911508) have been reported previously.^10,19^ Each participating centre obtained ethical approval from its institutional review board or ethics committee, and all participants provided written informed consent. Participants were randomized 1:1 to catheter ablation or drug therapy via a centralized, stratified system. Ablation involved pulmonary vein isolation in all cases, with adjunctive strategies permitted at investigator discretion. The primary goal was elimination of AF. Drug therapy began with rate control, with rhythm control or cardioversion allowed as needed. According to the CABANA protocol, patients with a CHA₂DS₂-VASc score ≥ 2 were typically continued on oral anticoagulation post-ablation, with discontinuation left to physician discretion. Full intervention details are available in the primary publication.^10^ This trial received funding from the National Institutes of Health and was carried out in accordance with the Declaration of Helsinki and CONSORT guidelines.

Participants in the CABANA trial were eligible if they met the following criteria: (i) aged ≥65 years, or <65 years with at least one stroke risk factor, including heart failure, diabetes, hypertension, previous stroke, or other cardiovascular disease; (ii) had experienced at least two episodes of paroxysmal AF or one episode of persistent AF within the previous 6 months; and (iii) had no prior AF catheter ablation and had not failed two or more antiarrhythmic medications.

### Stratification strategy based on comorbidity burden

In this analysis, comorbidity burden was stratified specifically for the CABANA cohort, without the intention of establishing a universally applicable threshold for high multimorbidity in the broader AF population. We assessed comorbidity burden based on the 15 predefined comorbidities collected according to the CABANA trial protocol: sleep apnoea, oesophageal disease, cancer, coronary artery disease, diabetes mellitus, valve disease, hypertension, hypercholesterolaemia, chronic lung disease, congestive heart failure, history of stroke or transient ischaemic attack, peripheral thromboembolic events, renal disease, thyroid disease, and cardiomyopathy. All comorbidity information was prospectively obtained via site-reported electronic medical records, using predefined fields in the trial electronic case report form. While not all comorbidities had formally standardized operational definitions, consistent data entry procedures across participating centres ensured data integrity. Detailed definitions are provided in Supplementary material online, *Table S1*.

To define an appropriate threshold for categorizing comorbidity burden, we considered both the distribution of comorbidities in the cohort and the number of observed outcome events under different cut-offs. Thresholds that encompassed a large proportion of patients offered limited discrimination between groups, whereas higher thresholds, requiring a greater number of comorbidities, reduced statistical power due to fewer outcome events. This approach aimed to ensure a meaningful separation of patient subgroups while preserving the robustness of outcome comparisons.

As a second analytical strategy to support our categorization, we modelled the number of comorbidities as a continuous variable and examined its interaction with treatment assignment using restricted cubic splines (RCS) in a Cox proportional hazards framework for the primary outcome. Hazard ratios (HRs) for catheter ablation vs. drug therapy were estimated across the comorbidity continuum, with 95% confidence intervals (CIs) derived from 1000 bootstrap replicates to enhance estimation stability, particularly at the distributional extremes. The resulting curve was examined to identify regions where the HRs and CIs trended below or around 1.0, providing further empirical support for the chosen stratification approach.

### Study outcomes

The primary outcome, aligned with the definition used in the CABANA trial, was a composite of all-cause mortality, disabling stroke, serious bleeding, or cardiac arrest. Disabling stroke (including intracranial haemorrhage) was defined as an irreversible physical impairment corresponding to a modified Rankin Scale score of ≥2. Serious bleeding was defined as bleeding associated with haemodynamic compromise requiring surgical intervention or transfusion of ≥3 units of blood. An independent clinical events committee, blinded to treatment allocation, adjudicated all primary outcome components based on pre-specified definitions. The secondary outcomes included (i) cardiovascular hospitalization and (ii) a composite of all-cause mortality or cardiovascular hospitalization.

Atrial fibrillation recurrence and AF-specific quality of life (QoL) were evaluated as additional exploratory outcomes in respective subsets. Atrial fibrillation recurrence was assessed in a subset of patients using the ECG monitoring system Medicomp (Medicomp, Inc. Melbourne, Florida),^20^ with data recorded in the ‘MEDICOMP.csv’ file. Recurrence event was defined as a symptomatic episode of AF, atrial flutter, or atrial tachycardia lasting ≥30 s, occurring after a 90-day post-treatment blanking period. Quality of life was measured using the Mayo Atrial Fibrillation Symptom Inventory (MAFSI) frequency score^19^ and was assessed in a subset of patients with complete data from baseline to 24 months.

### Statistical analysis

Some variables contained a small proportion of missing data (see Supplementary material online, *Table S2*) and were imputed using the ‘mice’ package in R, with five imputations and default methods. All continuous variables were non-normally distributed, as confirmed by normality testing. Accordingly, continuous variables were summarized as median with interquartile range (IQR) and compared using the Mann–Whitney *U* test. Categorical variables were presented as counts with percentages and compared using the Pearson *χ*² test or Fisher’s exact test.

Event rates in the catheter ablation and drug therapy groups were calculated separately within each level of comorbidity burden and expressed as events per 100 person-years. Rate differences with 95% CIs were estimated using the Wald method for independent Poisson rates, and corresponding *P* values were derived from two-proportion tests weighted by person-years. Time-to-first-event analyses for the primary outcome, its individual components, and each secondary outcome were conducted using Cox proportional hazards models, based on the intention-to-treat (ITT) principle. To aid interpretation of treatment-by-comorbidity interactions, we also estimated treatment effects in the overall cohort within the ITT principle. Each model was adjusted for AF type and CHA₂DS₂-VASc score. Proportional hazards assumptions were verified using Schoenfeld residuals and found to be satisfied for all models. Adjusted HRs (aHRs) with 95% CIs and interaction *P* values were reported. An interaction term between treatment group and comorbidity burden was additionally included, and the interaction *P* value was reported. Subgroup analyses assessed treatment effects by age (<60 vs. ≥60 years), sex, AF type (paroxysmal vs. non-paroxysmal), and CHA₂DS₂-VASc score (low vs. high), with reporting corresponding interaction *P* values.

A per-protocol analysis was conducted to evaluate treatment effects under conditions of adherence. The drug therapy group included all patients randomized to drug therapy, with follow-up censored at the time of crossover to catheter ablation. The ablation group comprised patients who underwent the procedure within 6 months of randomization. An as-treated analysis was additionally performed using the Cox model with catheter ablation included as a time-dependent covariate. Both analyses used the same Cox modelling approach as in the ITT analysis.

In the QoL analysis, linear mixed-effects models were used (via the ‘lme4’ and ‘lmerTest’ packages in R) to assess the effect of treatment (catheter ablation vs. drug therapy) on symptom progression, stratified by comorbidity burden. For each stratum, time, treatment, and their interaction were modelled as fixed effects, with a random intercept for each patient to account for within-subject correlation. Estimated marginal means (EMMs), adjusted mean differences, 95% CIs, and *P* values were obtained using the ‘emmeans’ package. Results were visualized as changes in EMMs over time by treatment group within each comorbidity stratum.

Statistical analyses were performed in R (R Foundation for Statistical Computing, Vienna, Austria), and a *P* < 0.05 (two-sided) was deemed statistically significant.

## Results

### Determination of comorbidity burden threshold

**Figure 1:**
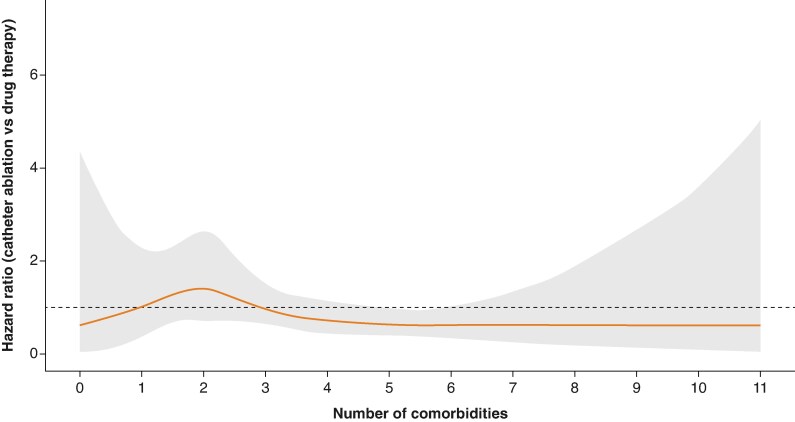
Association between comorbidity burden and the relative treatment effect of catheter ablation vs. drug therapy. Hazard ratios for the primary outcome comparing catheter ablation with drug therapy were estimated using a Cox proportional hazards model with RCS for the number of comorbidities. The orange line represents HRs across the comorbidity continuum; the shaded grey area indicates 95% CIs based on 1000 bootstrap replications. The dashed line at HR = 1.0 marks the null effect, with HRs declining and stabilizing below this threshold beyond a comorbidity count of 4.

A comorbidity threshold of ≥4 was selected to define high burden based on multiple considerations. As shown in Supplementary material online, *Table S3*, this cut-off captured 33.4% of patients and corresponded to higher event rates. Supplementary material online, *Table S4* further demonstrated that, among the thresholds tested, ≥4 yielded the highest statistical power under comparable effect assumptions. Additionally, RCS modelling (*Figure 1*) revealed a non-linear association between comorbidity burden and the relative treatment effect of ablation. Due to sparse data and wider CIs at both extremes, interpretation focused on the central range (approximately 2–6), where most patients were concentrated. Within this region, the HR declined progressively with increasing comorbidity count and stabilized below 1.0 beyond a count of 4. This pattern supports treatment effect modification across the comorbidity burden and reinforces the empirical rationale for stratifying patients at this threshold.

### Baseline characteristics

In the original CABANA cohort, 736 patients were identified as having a high comorbidity burden, with a median age of 68.0 years (IQR: 63.0–73.0), and 494 (67.1%) were male. In contrast, 1468 patients had a low comorbidity burden [median age 67.0 years (IQR: 61.0–71.0)]; 892 (60.7%) were males. The median follow-up duration in this group was 3.9 years (IQR: 2.4–5.1).

Patients with high comorbidity burden had higher CHA₂DS₂-VASc scores compared to those with low comorbidity burden [median 4.0 (IQR: 3.0–5.0) vs. 2.0 (IQR: 1.0–3.0)]. *Table 1* presents baseline characteristics stratified by comorbidity burden and treatment strategy. Within each stratum, baseline characteristics were largely comparable between the catheter ablation and drug therapy groups. Pulse rate was slightly lower in the catheter ablation group among patients with low comorbidity burden. In the high comorbidity burden group, cardiomyopathy was more common in the drug therapy group (26.4% vs. 17.8%), while other comorbidities showed no significant between-group differences.

**Table 1:** Baseline characteristics of patients stratified by comorbidity burden and treatment strategy

	Low comorbidity burden (<4 comorbidities)			High comorbidity burden (≥4 comorbidities)		
	Catheter ablation	Drug therapy	*P* value	Catheter ablation	Drug therapy	*P* value
*n*	743	725		365	371	
Age, years	67.0 (61.0, 71.0)	67.0 (61.0, 71.0)	0.774	68.0 (63.0, 73.0)	68.0 (62.0, 72.0)	0.130
Male, *n* (%)	455 (61.2)	436 (60.1)	0.705	240 (65.8)	254 (68.5)	0.481
Body mass index, kg/m^2^	29.1 (26.1, 33.0)	29.3 (26.0, 33.5)	0.702	32.0 (28.1, 36.3)	32.1 (28.5, 36.7)	0.371
Systolic blood pressure, mmHg	128.0 (120.0, 139.0)	130.0 (120.0, 140.0)	0.413	130.0 (120.0, 140.0)	128.0 (116.0, 140.0)	0.155
Diastolic blood pressure, mmHg	80.0 (71.0, 84.0)	80.0 (71.0, 85.0)	0.461	76.0 (69.0, 84.0)	76.0 (70.0, 83.0)	0.908
Pulse, b.p.m.	72.0 (63.0, 85.0)	74.0 (65.0, 86.0)	0.033	74.0 (63.0, 86.0)	75.0 (63.0, 86.0)	0.984
Race, *n* (%)			0.972			0.676
White	681 (91.7)	666 (91.9)		342 (93.7)	345 (93.0)	
Black or African American	20 (2.7)	20 (2.8)		19 (5.2)	19 (5.1)	
Other	42 (5.7)	39 (5.4)		4 (1.1)	7 (1.9)	
NYHA class, *n* (%)			0.824			0.160
<2	497 (66.9)	480 (66.2)		230 (63.0)	213 (57.4)	
≥2	246 (33.1)	245 (33.8)		135 (37.0)	158 (42.6)	
AF severity, *n* (%)			0.687			0.288
Class 0	73 (9.8)	87 (12.0)		32 (8.8)	32 (8.6)	
Class I	125 (16.8)	118 (16.3)		42 (11.5)	55 (14.8)	
Class II	258 (34.7)	243 (33.5)		95 (26.0)	113 (30.5)	
Class III	253 (34.1)	249 (34.3)		152 (41.6)	134 (36.1)	
Class IV	34 (4.6)	28 (3.9)		44 (12.1)	37 (10.0)	
AF type, *n* (%)			0.863			0.432
Paroxysmal	330 (44.4)	319 (44.0)		140 (38.4)	158 (42.6)	
Persistent	335 (45.1)	335 (46.2)		189 (51.8)	183 (49.3)	
Long-standing persistent	78 (10.5)	71 (9.8)		36 (9.9)	30 (8.1)	
CHA_2_DS_2_-VASc score	2.00 (1.00, 3.00)	2.00 (2.00, 3.00)	0.808	4.00 (3.00, 5.00)	3.00 (3.00, 5.00)	0.426
Anticoagulation status, *n* (%)						
Warfarin	293 (39.4)	299 (41.2)	0.489	158 (43.3)	163 (43.9)	0.882
DOACs^a^	117 (15.7)	111 (15.3)	0.829	70 (19.2)	59 (15.9)	0.247
Comorbidities, *n* (%)						
Sleep apnoea	94 (12.7)	95 (13.1)	0.857	168 (46.0)	151 (40.7)	0.166
Oesophageal disease	55 (7.4)	66 (9.1)	0.276	131 (35.9)	135 (36.4)	0.949
Cancer	23 (3.1)	26 (3.6)	0.705	37 (10.1)	43 (11.6)	0.607
Coronary artery disease	52 (7.0)	54 (7.4)	0.817	156 (42.7)	162 (43.7)	0.858
Diabetes mellitus	112 (15.1)	103 (14.2)	0.692	168 (46.0)	178 (48.0)	0.648
Valve disease	60 (8.1)	46 (6.3)	0.238	79 (21.6)	80 (21.6)	1.000
Hypertension	538 (72.4)	550 (75.9)	0.147	338 (92.6)	350 (94.3)	0.421
Hypercholesterolaemia	238 (32.0)	207 (28.6)	0.163	285 (78.1)	286 (77.1)	0.814
Chronic lung disease	34 (4.6)	38 (5.2)	0.639	67 (18.4)	58 (15.6)	0.376
Congestive heart failure	53 (7.1)	48 (6.6)	0.776	121 (33.2)	115 (31.0)	0.584
History of CVA/TIA	54 (7.3)	46 (6.3)	0.550	63 (17.3)	57 (15.4)	0.551
Peripheral thromboembolic events	17 (2.3)	15 (2.1)	0.914	24 (6.6)	34 (9.2)	0.243
Renal disease	0 (0.0)	0 (0.0)	–	5 (1.4)	7 (1.9)	0.793
Thyroid disease	77 (10.4)	59 (8.1)	0.167	80 (21.9)	84 (22.6)	0.883
Cardiomyopathy	34 (4.6)	23 (3.2)	0.209	65 (17.8)	98 (26.4)	0.006

All continuous variables are presented as median (interquartile range) due to non-normal distributions; categorical variables are presented as number (percentage). Group comparisons were performed using the Kruskal–Wallis test for continuous variables and the *χ*² test for categorical variables.

AF, atrial fibrillation; CVA, cerebral vascular accident; DOAC, direct oral anticoagulant; NYHA, New York Heart Association; TIA, transient ischaemic attack.

^a^DOACs included dabigatran, rivaroxaban, apixaban, and edoxaban.

### Overall intention-to-treat (as-randomized) treatment comparisons

In the overall cohort, ITT analysis showed that catheter ablation was associated with a numerically lower risk of the primary outcome compared to drug therapy [87 events (7.9%) vs. 98 (8.9%); 2.1 vs. 2.4 per 100 person-years; aHR: 0.85, 95% CI: 0.63–1.13; *P* = 0.254], although this difference was not significant. No significant differences were observed for individual components of the primary outcome. However, catheter ablation significantly reduced the risk of cardiovascular hospitalization (aHR: 0.84, 95% CI: 0.75–0.95; *P* = 0.005) and the composite of all-cause mortality or cardiovascular hospitalization (aHR: 0.83, 95% CI: 0.74–0.93; *P* = 0.001). Full results are provided in Supplementary material online, *Table S5*.

### Intention-to-treat (as-randomized) treatment comparisons by comorbidity burden

**Figure 2:**
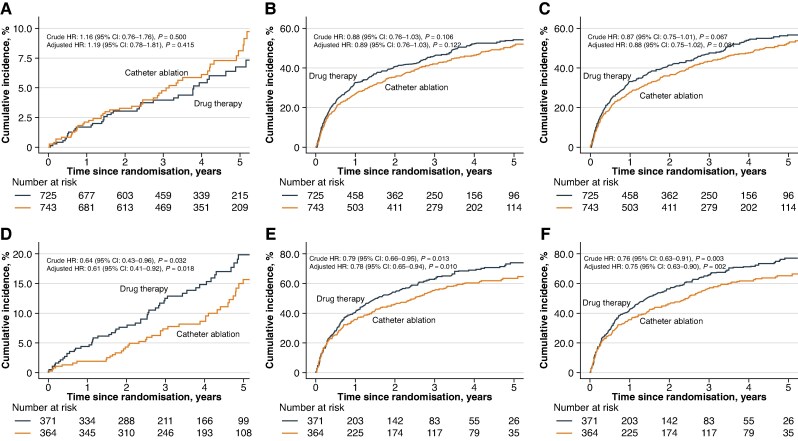
Kaplan–Meier curves comparing clinical outcomes between catheter ablation and drug therapy by comorbidity burden. Panels (*A*)–(*C*) show outcomes in patients with low comorbidity burden (<4 comorbidities), and panels (*D*)–(*F*) show outcomes in those with high comorbidity burden (≥4 comorbidities). Panels (*A*) and (*D*) present the primary composite outcome (all-cause mortality, disabling stroke, serious bleeding, or cardiac arrest); panels (*B*) and (*E*) show cardiovascular hospitalization; and panels (*C*) and f show the composite of all-cause mortality or cardiovascular hospitalization. Adjusted HRs and 95% CIs were estimated using Cox proportional hazards models adjusted for age, sex, atrial fibrillation type and CHA₂DS₂-VASc score. CI, confidence interval; HR, hazard ratio.

In the ITT treatment comparisons, clinical outcomes differed by comorbidity burden (*Table 2* and *Figure 2*). Among patients with high comorbidity burden, catheter ablation was associated with a lower incidence of the primary composite outcome compared to drug therapy [40 events (11.0%) vs. 57 events (15.4%); 2.8% vs. 4.4% per 100 person-years; aHR: 0.61, 95% CI: 0.41–0.92, *P* = 0.018]. Among those with low comorbidity burden, no significant difference was observed [47 events (6.3%) vs. 41 events (5.7%); 1.7% vs. 1.5% per 100 person-years; aHR: 1.19, 95% CI: 0.78–1.81, *P* = 0.415]. There was a significant interaction between treatment effect and comorbidity burden (interaction *P* = 0.027).

**Table 2:** Clinical outcomes across comorbidity burden in the ITT analysis

		Catheter ablation		Drug therapy		Rate difference (95% CI) (%)^a^	*P* value^a^	aHR (95% CI)^b^	*P* value^b^	Interaction *P* value
		Events, *n* (%)	Rate (%/year)	Events, *n* (%)	Rate (%/year)					
Primary outcome^c^	Low	47 (6.3)	1.7	41 (5.7)	1.5	0.21 (−0.45, 0.87)	0.602	1.19 (0.78, 1.81)	0.415	0.027
	High	40 (11.0)	2.8	57 (15.4)	4.4	−1.55 (−2.97, −0.12)	0.037	0.61 (0.41, 0.92)	0.018	
Components of primary outcome										
All-cause mortality	Low	29 (3.9)	1.0	27 (3.7)	1.0	0.06 (−0.46, 0.58)	0.913	1.10 (0.65, 1.86)	0.721	0.202
	High	28 (7.7)	1.9	37 (10.0)	2.7	−0.76 (−1.87, 0.35)	0.217	0.69 (0.42, 1.12)	0.133	
Disabling stroke	Low	3 (0.4)	0.1	4 (1.1)	0.1	−0.04 (−0.22, 0.15)	0.993	0.74 (0.17, 3.33)	0.697	–
	High	0 (0.0)	0.0	3 (0.8)	0.2	−0.22 (−0.46, 0.03)	0.229	–	–	
Cardiac arrest	Low	4 (0.5)	0.1	4 (1.1)	0.1	0.00 (−0.20, 0.20)	1.000	1.03 (0.26, 4.14)	0.968	0.363
	High	3 (0.4)	0.2	7 (1.9)	0.5	−0.31 (−0.75, 0.14)	0.290	0.41 (0.11, 1.59)	0.197	
Serious bleeding	Low	20 (2.7)	0.7	12 (1.7)	0.4	0.29 (−0.11, 0.68)	0.215	1.74 (0.85, 3.57)	0.130	0.015
	High	14 (3.8)	1.0	24 (6.5)	1.8	−0.83 (−1.71, 0.06)	0.090	0.52 (0.27, 1.00)	0.050	
Secondary outcome										
CV hospitalization	Low	325 (43.7)	17.4	344 (47.4)	20.4	−3.07 (−5.93, −0.20)	0.022	0.89 (0.76, 1.03)	0.122	0.331
	High	209 (57.3)	26.2	239 (64.4)	35.7	−9.53 (−15.28, −3.78)	<0.001	0.78 (0.65, 0.94)	0.010	
All-cause mortality/CV hospitalization	Low	337 (45.4)	18.0	361 (49.8)	21.4	−3.44 (−6.36, −0.51)	0.011	0.88 (0.75, 1.02)	0.081	0.235
	High	215 (58.9)	26.9	254 (68.5)	38.0	−11.02 (−16.91, −5.13)	<0.001	0.75 (0.63, 0.90)	0.002	

‘High’ indicates ≥4 comorbidities; ‘Low’ indicates <4 comorbidities, defined based on baseline comorbidity count in the CABANA trial.

AF, atrial fibrillation; CI, confidence interval; CV, cardiovascular; aHR, adjusted hazard ratio; ITT, intention-to-treat.

^a^Event rates are expressed as events per 100 person-years. Rate differences with 95% confidence intervals were calculated using the Wald method for independent Poisson rates. Corresponding *P* values were obtained from two-proportion tests weighted by person-years.

^b^Adjusted HRs and corresponding 95% CIs were estimated using Cox proportional hazards models adjusting for age, sex, AF type, and CHA_2_DS_2_-VASc score. The drug therapy group was used as the reference category for all comparisons.

^c^The primary outcome was a composite endpoint including all-cause mortality, disabling stroke, cardiac arrest, or serious bleeding events.

For individual components of the primary outcome, including all-cause mortality, disabling stroke, cardiac arrest, and serious bleeding, no statistically significant differences were observed in either comorbidity subgroup, although a borderline trend towards reduced serious bleeding was observed in the high comorbidity burden group (aHR: 0.52, 95% CI: 0.27–1.00, *P* = 0.050), with a significant statistical interaction (interaction *P* = 0.015).

For the secondary outcomes in patients with high comorbidity burden, catheter ablation was associated with a significantly lower risk of both cardiovascular hospitalization (aHR: 0.78, 95% CI: 0.65–0.94, *P* = 0.010) and the composite of all-cause mortality or cardiovascular hospitalization (aHR: 0.75, 95% CI: 0.63–0.90, *P* = 0.002). These associations were not observed in the low comorbidity burden group, and no significant statistical interactions were seen.

### Subgroup analysis in patients with high comorbidity burden (intention-to-treat)

Subgroup analyses of the primary outcome and secondary outcomes showed consistent trends favouring catheter ablation across most strata in patients with high comorbidity burden (see Supplementary material online, *Figure S1*), with no significant statistical interactions observed.

### Per-protocol treatment comparisons by comorbidity burden

In the per-protocol treatment comparisons (see Supplementary material online, *Table S6*), catheter ablation was associated with a significantly lower risk of the primary outcome in patients with high comorbidity burden (aHR: 0.56, 95% CI: 0.36–0.87, *P* = 0.009), while no significant difference seen in those with lower comorbidity burden (aHR: 0.96, 95% CI: 0.61–1.49, *P* = 0.846); however, there was no statistically significant interaction (interaction *P* = 0.106).

Among components of the primary outcome, catheter ablation was associated with lower all-cause mortality in patients with high comorbidity burden (aHR: 0.57, 95% CI: 0.33–0.96, *P* = 0.035), but not in the low comorbidity burden group. For the composite outcome of all-cause mortality or cardiovascular hospitalization, catheter ablation was also associated with a significantly lower risk in the high comorbidity burden group (aHR: 0.80, 95% CI: 0.66–0.96, *P* = 0.018), while no significant benefit was observed in the lower comorbidity burden group.

Other outcomes showed numerically lower event rates with ablation in the high comorbidity burden group, though the differences did not reach statistical significance.

### As-treated treatment comparisons by comorbidity burden

In the as-treated analysis (see Supplementary material online, *Table S7*), catheter ablation was linked to a significantly lower risk of the primary outcome among patients with high comorbidity burden (aHR: 0.59, 95% CI: 0.38–0.90, *P* = 0.015), whereas no significant benefit was seen in those with low comorbidity burden (aHR: 1.08, 95% CI: 0.70–1.65, *P* = 0.731), with significant interaction (interaction *P* = 0.049).

A significant interaction was noted for serious bleeding (interaction *P* = 0.007), with an increased risk in patients with low comorbidity burden (aHR: 2.04, 95% CI: 0.99–4.20, *P* = 0.053) and a reduced risk among those with high comorbidity burden (aHR: 0.49, 95% CI: 0.24–1.00, *P* = 0.051).

For other outcomes, including all-cause mortality, disabling stroke, cardiac arrest, and cardiovascular hospitalization, no consistent or statistically significant differences were observed across comorbidity strata.

### Atrial fibrillation recurrence (intention-to-treat)

Among 1351 patients with available CABANA Box records during the 3-month blanking period following randomization, catheter ablation was consistently associated with a significantly lower risk of AF recurrence across comorbidity strata (see Supplementary material online, *Table S8*). The aHR was 0.51 (95% CI: 0.42–0.61; *P* < 0.001) in the low comorbidity burden group and 0.60 (95% CI: 0.48–0.74; *P* < 0.001) in the high comorbidity burden group.

### Quality of life analysis (intention-to-treat)

**Figure 3:**
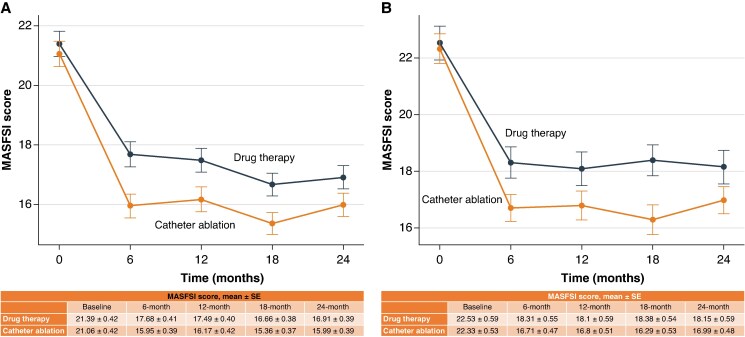
Longitudinal changes in quality of life (MASFSI score) following catheter ablation vs. drug therapy stratified by comorbidity burden. Panel (*A*) shows estimated mean MASFSI frequency scores in patients with low comorbidity burden (<4 comorbidities), and panel (*B*) shows those with high comorbidity burden (≥4 comorbidities). Scores were analysed using linear mixed-effects models with time, treatment, and time-by-treatment interaction as fixed effects and a random intercept for each patient. Error bars represent 95% CIs. CI, confidence interval; MAFSI, Mayo Atrial Fibrillation Symptom Inventory.

At baseline, MASFSI frequency scores were comparable between the two treatment arms in both comorbidity strata (*Figure 3*). The greatest reduction in symptom burden occurred by 6 months following catheter ablation, with an adjusted mean difference of −1.73 (95% CI: −2.43 to −1.03) in the low comorbidity group and −1.60 (95% CI: −2.36 to −0.84) in the high comorbidity group, compared to drug therapy. Thereafter, scores remained consistently lower in the ablation group.

At 18 months, symptom improvement remained more pronounced among patients with high comorbidity burden, with an adjusted mean difference of −2.09 (95% CI: −3.12 to −1.07), compared to −1.30 (95% CI: −2.16 to −0.44) in the low-burden group. At 24 months, the corresponding differences were −1.17 (95% CI: −2.29 to −0.06) and −0.93 (95% CI: −1.90 to 0.03), respectively.

## Discussion

This sub-analysis of the CABANA trial demonstrates that the therapeutic efficacy of catheter ablation for AF may differ according to comorbidity burden. In the overall cohort, ITT analysis showed a numerically lower risk of the primary composite outcome with catheter ablation compared to drug therapy, although this difference did not reach statistical significance, consistent with the results of the original CABANA publication.^10^ The key findings of our comorbidity-stratified analyses are as follows: (i) catheter ablation was associated with significantly lower risks of the primary and secondary outcomes in patients with high comorbidity burden. In contrast, no statistically significant differences were observed in the low-burden group, where the ablation arm showed a numerically higher event rate for the primary outcome and lower rates for secondary outcomes compared to drug therapy. These patterns, together with a significant interaction for the primary outcome, suggest potential effect modification by comorbidity burden. (ii) Catheter ablation was consistently associated with lower AF recurrence across comorbidity strata. (iii) Catheter ablation was associated with greater improvements in quality of life compared to drug therapy across both comorbidity strata, with more pronounced long-term differences observed in patients with high comorbidity burden. These findings highlight the potential value of tailoring rhythm management strategies according to comorbidity burden.

A recent sub-analysis of the EAST-AFNET4 trial stratified patients by CHA₂DS₂-VASc score and found that early rhythm control (antiarrhythmic drugs, catheter ablation, or cardioversion) significantly reduced adverse cardiovascular outcomes compared to rate control–based usual care in patients with CHA₂DS₂-VASc ≥4, but not in those with lower scores.^21^ However, the study did not directly compare ablation with antiarrhythmic drugs, limiting conclusions on the relative efficacy of specific rhythm control strategies, a key consideration for clinical decision-making. Moreover, the CHA₂DS₂-VASc score includes age and sex and captures a narrow spectrum of cardiovascular comorbidities, as it was originally developed to estimate stroke risk in AF patients based on established thromboembolic predictors, making it an incomplete proxy for the broader multimorbidity seen in AF populations. Our study extends prior evidence by comparing ablation with antiarrhythmic therapy in multimorbid patients, considering a wider spectrum of comorbidities and identifying heterogeneity in treatment response through burden- and phenotype-based analyses.

While 2023 AHA/ACC/HRS guidelines have primarily supported first-line ablation in younger, healthier patients with fewer comorbidities (Class I recommendation) and recommended it more selectively for older individuals with multiple comorbidities (Class IIa),^8^ our findings broaden the evidence base and support its use in the latter population, who have been underrepresented in prior trials.^10,14–18,22,23^ In CABANA participants with ≥4 comorbidities, who represented a predominantly older population (83.8% aged ≥60 years), we found that these individuals, although typically receiving a lower guideline recommendation for catheter ablation (Class IIa rather than Class I), also derived clear clinical benefit from the procedure. In this group, catheter ablation was associated with significantly better outcomes than drug therapy, including reductions in all-cause mortality, disabling stroke, cardiac arrest, serious bleeding, and cardiovascular hospitalization. These findings were consistent in both ITT and per-protocol analyses, reinforcing their robustness. Furthermore, in patients aged ≥60 years, subgroup analysis confirmed that catheter ablation yielded superior primary and secondary outcomes compared to drug therapy. Although the primary CABANA trial showed no significant difference between ablation and drug therapy in the overall population,^10^ our findings highlight multimorbidity as a key modifier of treatment response.

Among patients with lower comorbidity burden, catheter ablation was not associated with a reduced risk of the primary outcome compared to drug therapy. However, this group still experienced improvements in other clinically relevant measures, including significant reductions in AF recurrence, numerically lower cardiovascular hospitalization rates, and enhanced AF-related quality of life. These findings suggest that even in the absence of a primary outcome benefit, ablation may still confer symptomatic and functional advantages in lower-risk populations, aligning with guideline emphasis on symptom relief and rhythm control in appropriately selected patients.

Importantly, catheter ablation is fundamentally a symptom-control intervention, aligning with the ‘B’ component (better symptom control) of the ‘Atrial fibrillation Better Care’ (ABC) pathway. Prior studies have demonstrated that the ABC pathway improves clinical outcomes in AF patients with multimorbidity,^24–26^ supporting its applicability in complex clinical settings. Situating our findings within this established framework strengthens their clinical interpretation and highlights the relevance of symptom-focused strategies in the broader context of integrated AF care.

The differential treatment effect observed across comorbidity strata is biologically plausible and likely reflects variation in underlying pathophysiological mechanisms. In multimorbid patients, AF may accelerate deterioration via atrioventricular dyssynchrony, neurohormonal activation, atrial remodelling, autonomic dysregulation, and systemic inflammation.^27^ These pathophysiologic mechanisms may amplify haemodynamic compromise and end-organ dysfunction. Effective rhythm control through catheter ablation could interrupt this cycle, reduce systemic stress, and promote clinical stabilization.^22^

Notably, catheter ablation was associated with a lower incidence of major bleeding in patients with high comorbidity burden, a pattern not observed in those with fewer comorbidities, where bleeding risk may have been increased. This differential effect may partly reflect the higher body mass index (BMI) observed in the high-burden group [median 32.0 kg/m² (IQR: 28.4–36.6) vs. 29.2 kg/m² (26.0–33.2)]. Prior evidence suggests that individuals with higher BMI may experience a lower risk of bleeding while on oral anticoagulation,^28,29^ potentially due to pharmacokinetic differences such as increased volume of distribution and altered drug clearance.^30^ Given that anticoagulation was protocol-mandated following catheter ablation in CABANA, standardized dosing may not have accounted for interindividual variability in body mass. This may have conferred a relative pharmacokinetic advantage to higher BMI patients, contributing to the reduced bleeding risk observed in this group, whereas lower BMI individuals may have experienced heightened anticoagulant exposure and bleeding susceptibility.

### Limitations

This study has several limitations. First, this was a *post hoc* analysis that was not pre-specified in the original trial protocol. As such, the findings may be subject to bias or residual confounding and should be interpreted as exploratory and hypothesis-generating. Second, although the present analysis focused on comorbidity burden as a potential modifier of treatment effect, the CABANA trial was not stratified by comorbidity status at randomization. Despite the generally balanced baseline characteristics between ablation and drug therapy groups within each comorbidity stratum, unmeasured confounding may still exist, and the results should be interpreted with caution. Third, treatment crossovers occurred during the trial, particularly from the drug therapy arm to ablation, which may have attenuated the observed treatment effects in the ITT analysis. To address this, we performed a pre-protocol analysis adjusting for treatment crossovers; however, such analyses are inherently subject to selection bias. Fourth, information on AF recurrence and symptom scores was only available in subsets of the trial population, which may limit the generalisability of these findings. Fifth, comorbidity burden was defined based on comorbid conditions collected according to the CABANA trial protocol. While these conditions represent common and clinically relevant comorbidities in AF patients, it is possible that other important comorbidities were not captured, potentially leading to an underestimation of the total comorbidity burden. Sixth, the definition of high comorbidity burden (≥4 conditions) may limit generalisability, as this group represented a smaller subset of the CABANA population. While the threshold was selected using a data-driven approach, considering comorbidity distribution and modelling interaction effects via RCS analysis, any *post hoc* cut-off remains somewhat arbitrary and may not reflect a universally applicable definition of multimorbidity in AF. Further prospective validation is warranted in independent populations. Seventh, comorbidity data were collected via site-reported clinical diagnoses using predefined fields in the trial electronic case report form, but without central adjudication, severity grading, or uniformly standardized operational definitions. This may introduce heterogeneity and misclassification, particularly for conditions such as oesophageal or renal disease, where definitions were vague or site-dependent. These limitations underscore the need for future RCTs with centrally adjudicated, standardized definitions and structured multimorbidity assessment. Eighth, the limited sample size precluded more refined stratification of comorbidity burden. Validation in larger cohorts is warranted to assess treatment gradients across the full comorbidity spectrum. Finally, CABANA recruited patients between 2009 and 2016, prior to the widespread implementation of newer ablation strategies and early rhythm control approaches.^31,32^ These temporal differences may limit the direct applicability of our findings to contemporary clinical practice, underscoring the need for validation in current real-world AF populations.

## Conclusions

In this *post hoc* analysis of the CABANA trial, catheter ablation was associated with more favourable clinical outcomes and improved quality of life compared to drug therapy in AF patients with a high comorbidity burden. These findings suggest that individuals with multiple comorbidities, who have been underrepresented in prior trials, may also derive meaningful clinical benefit from catheter ablation. Consideration of comorbidity burden may help guide more individualized rhythm management strategies in routine practice.

## Supplementary Material

euaf292_Supplementary_Data

## Data Availability

CABANA trial data were accessed via the Biologic Specimen and Data Repository Information Coordinating Center of the National Heart, Lung, and Blood Institute under a data use agreement. The authors did not participate in the original trial. Access to the CABANA data can be requested via the BioLINCC website (https://biolincc.nhlbi.nih.gov).
