# Supplementary material for: Association between comorbidity burden and outcomes of catheter ablation vs. medical therapy for atrial fibrillation: insights from the CABANA trial
Source: Europace. 2025 Nov 10;27(12):euaf292. doi: 10.1093/europace/euaf292 (PMC12677905; doi:10.1093/europace/euaf292)
Supplement: euaf292_Supplementary_Data [file euaf292_supplementary_data.docx]

Supplementary Table S1. Comorbidity definitions according to the electronic Case Report Form of CABANA trial.

| Comorbidity | Definition |
| --- | --- |
| Sleep apnea | No formal definition provided |
| Esophageal disease | No formal definition provided |
| Cancer | No formal definition provided |
| Coronary artery disease | Defined as including MI, CABG, PCI, or previously diagnosed CAD |
| Diabetes mellitus | Defined as fasting plasma glucose over 126 mg/dL |
| Valve disease | Defined as the presence of valvular stenosis or insufficiency (regurgitation) |
| Hypertension | Defined as systolic blood pressure over 140 mmHg or diastolic blood pressure over 90 mmHg |
| Hypercholesterolemia | No formal definition provided |
| Chronic lung disease | No formal definition provided |
| Congestive heart failure | No formal definition provided |
| History of CVA/TIA | No formal definition provided |
| Peripheral thromboembolic events | No formal definition provided |
| Renal disease | Defined as the presence of renal disease and/or current or anticipated dialysis |
| Thyroid disease | No formal definition provided |
| Cardiomyopathy | Defined as including hypertrophic (excluding obstructive cases with over 30 mmHg gradient), dilated, restrictive, or tachycardia-induced forms |

Abbreviations: CABG, coronary artery bypass grafting; CAD, coronary artery disease; CVA, cerebrovascular accident; MI, myocardial infarction; PCI, percutaneous coronary intervention; TIA, transient ischemic attack.

Supplementary Table S2. Proportion of missing values for baseline variables.

| Variable | Missing, n (%) |
| --- | --- |
| Body mass index | 34 (1.54) |
| Systolic blood pressure | 24 (1.09) |
| Diastolic blood pressure | 28 (1.27) |
| Pulse | 16 (0.73) |
| Atrial fibrillation severity | 13 (0.59) |
| New York Heart Association Functional Classification | 18 (0.82) |
| Race | 2 (0.09) |
| Atrial fibrillation type | 1 (0.05) |

Supplementary Table S3. Distribution of composite outcome events by treatment and comorbidity count.

| Comorbidity Count | N (%) | Treatment | N (%) | Event, n (%) |
| --- | --- | --- | --- | --- |
| 0-2 | 1,021 (46.3) | Catheter ablation | 506 (49.6) | 26 (5.1) |
|  |  | Drug therapy | 515 (50.4) | 22 (4.3) |
|  |  |  |  |  |
| 3 | 447 (20.3) | Catheter ablation | 237 (53.0) | 21 (8.9) |
|  |  | Drug therapy | 210 (47.0) | 19 (9.0) |
|  |  |  |  |  |
| 4 | 343 (15.6) | Catheter ablation | 174 (50.7) | 40 (11.0) |
|  |  | Drug therapy | 169 (49.3) | 57 (15.4) |
|  |  |  |  |  |
| ≥5 | 393 (17.8) | Catheter ablation | 191 (48.6) | 25 (13.1) |
|  |  | Drug therapy | 202 (51.4) | 33 (16.3) |

Supplementary Table S4. Statistical power estimates for different comorbidity thresholds.

| Comorbidity Threshold | N (%) | Treatment | N (%) | Event, n (%) | Estimated Power (%) |
| --- | --- | --- | --- | --- | --- |
| Low comorbidity (<3) | 1,020 (46.3) | Catheter ablation | 506 (49.6) | 26 (5.1) | 63.8 |
|  |  | Drug therapy | 515 (50.4) | 22 (4.3) |  |
| High comorbidity (≥3) | 1,184 (53.7) | Catheter ablation | 602 (50.8) | 86 (14.3) | 99.6 |
|  |  | Drug therapy | 581 (49.1) | 109 (18.8) |  |
|  |  |  |  |  |  |
| Low comorbidity (<4) | 1,467 (66.6) | Catheter ablation | 743 (50.6) | 47 (6.3) | 87.1 |
|  |  | Drug therapy | 725 (49.4) | 40 (5.5) |  |
| High comorbidity (≥4) | 736 (33.4) | Catheter ablation | 365 (49.6) | 65 (17.8) | 98.6 |
|  |  | Drug therapy | 371 (50.4) | 90 (24.3) |  |
|  |  |  |  |  |  |
| Low comorbidity (<5) | 1,811 (82.2) | Catheter ablation | 917 (50.6) | 87 (9.5) | 99.5 |
|  |  | Drug therapy | 894 (49.4) | 98 (11.0) |  |
| High comorbidity (≥5) | 393 (17.8) | Catheter ablation | 191 (48.6) | 25 (13.1) | 72.6 |
|  |  | Drug therapy | 202 (51.4) | 33 (16.3) |  |

Statistical power was calculated using the powerSurvEpi R package, specifically the powerCT.default() function, assuming a two-sided α = 0.05, an expected hazard ratio of 2.0, and event rates and sample sizes observed under each comorbidity threshold scenario.

Supplementary Table S5. Clinical outcomes of ITT analysis in the overall cohort.

|  | Catheter Ablation | | Drug Therapy | | Rate Difference  (95% CI) (%)^a^ | P Value^a^ | aHR (95% CI)^b^ | P Value^b^ |
| --- | --- | --- | --- | --- | --- | --- | --- | --- |
|  | Events, n (%) | Rate (%/year) | Events, n (%) | Rate (%/year) |  |  |  |  |
| Primary outcome^c^ | 87 (7.9) | 2.1 | 98 (8.9) | 2.4 | -0.33 (-0.98, 0.31) | 0.339 | 0.85 (0.63, 1.13) | 0.254 |
|  |  |  |  |  |  |  |  |  |
| Components of primary outcome |  |  |  |  |  |  |  |  |
| All-Cause mortality | 57 (5.1) | 1.3 | 64 (5.8) | 1.5 | -0.20 (-0.71, 0.31) | 0.468 | 0.86 (0.60, 1.23) | 0.402 |
|  |  |  |  |  |  |  |  |  |
| Disabling stroke | 3 (0.3) | 0.1 | 7 (0.6) | 0.2 | -0.10 (-0.24, 0.05) | 0.221 | 0.41 (0.11, 1.58) | 0.195 |
|  |  |  |  |  |  |  |  |  |
| Cardiac arrest | 7 (0.6) | 0.2 | 11 (1.0) | 0.3 | -0.10 (-0.30, 0.10) | 0.353 | 0.61 (0.23, 1.56) | 0.300 |
|  |  |  |  |  |  |  |  |  |
| Serious bleeding | 34 (3.1) | 0.8 | 36 (3.3) | 0.9 | -0.07 (-0.46, 0.33) | 0.811 | 0.90 (0.56, 1.44) | 0.663 |
|  |  |  |  |  |  |  |  |  |
| Secondary outcome |  |  |  |  |  |  |  |  |
| CV hospitalisation | 534 (48.2) | 20.0 | 583 (53.2) | 24.8 | -4.78 (-7.41, -2.15) | < 0.001 | 0.84 (0.75, 0.95) | 0.005 |
|  |  |  |  |  |  |  |  |  |
| All-cause mortality/CV hospitalisation | 552 (49.8) | 20.7 | 615 (56.1) | 26.1 | -5.46 (-8.15, -2.77) | < 0.001 | 0.83 (0.74, 0.93) | 0.001 |

^a^Event rates are expressed as events per 100 person-years. Rate differences with 95% confidence intervals were calculated using the Wald method for independent Poisson rates. Corresponding P values were obtained from two-proportion tests weighted by person-years.

^b^aHRs and corresponding 95% CIs were estimated using Cox proportional hazards models adjusting for age, sex, AF type and CHA_2_DS_2_-VASc score. The drug therapy group was used as the reference category for all comparisons.

^c^The primary outcome was a composite endpoint including all-cause mortality, disabling stroke, cardiac arrest or serious bleeding events.

Abbreviations: AF, atrial fibrillation; CI, confidence interval; CV, cardiovascular; aHR, adjusted hazard ratio; ITT, intention-to-treat.

Supplementary Table S6. Clinical outcomes across comorbidity burden (<4 vs. ≥4)^a^ by pre-protocol analysis.

|  |  | Events in Ablation, n (%) | Events in Drug, n (%) | aHR (95% CI)^b^ | P Value | Interaction P Value |
| --- | --- | --- | --- | --- | --- | --- |
| Primary outcome^c^ | Low | 40 (6.1) | 38 (5.2) | 0.96 (0.61, 1.49) | 0.846 | 0.106 |
|  | High | 35 (11.0) | 46 (12.4) | 0.56 (0.36, 0.87) | 0.009 |  |
|  |  |  |  |  |  |  |
| Components of primary outcome | | | | | | |
| All-Cause mortality | Low | 25 (3.8) | 26 (3.6) | 0.85 (0.49, 1.48) | 0.577 | 0.337 |
|  | High | 25 (7.9) | 32 (8.6) | 0.57 (0.33, 0.96) | 0.035 |  |
|  |  |  |  |  |  |  |
| Disabling stroke | Low | 3 (0.5) | 4 (0.6) | 0.59 (0.13, 2.66) | 0.494 | - |
|  | High | 0 (0.0) | 3 (0.8) | - | - |  |
|  |  |  |  |  |  |  |
| Cardiac arrest | Low | 3 (0.5) | 4 (0.6) | 0.67 (0.15, 3.04) | 0.603 | 0.371 |
|  | High | 2 (0.6) | 6 (1.6) | 0.27 (0.05, 1.38) | 0.116 |  |
|  |  |  |  |  |  |  |
| Serious bleeding | Low | 18 (2.8) | 10 (1.4) | 1.68 (0.77, 3.66) | 0.188 | 0.057 |
|  | High | 13 (4.1) | 17 (4.6) | 0.58 (0.28, 1.20) | 0.143 |  |
|  |  |  |  |  |  |  |
| Secondary outcome |  |  |  |  |  |  |
| CV hospitalisation | Low | 291 (44.6) | 336 (46.3) | 0.92 (0.78, 1.07) | 0.279 | 0.448 |
|  | High | 189 (59.4) | 235 (63.3) | 0.83 (0.68, 1.01) | 0.058 |  |
|  |  |  |  |  |  |  |
| All-cause mortality/CV hospitalisation | Low | 300 (46.0) | 353 (48.7) | 0.90 (0.77, 1.05) | 0.175 | 0.353 |
|  | High | 194 (61.0) | 250 (67.4) | 0.80 (0.66, 0.96) | 0.018 |  |

^a^Among patients with <4 comorbidities (“Low”), 1377 individuals were included (652 in the catheter ablation group and 725 in the drug therapy group); among those with ≥4 comorbidities (“High”), 689 individuals were included (318 in the catheter ablation group and 371 in the drug therapy group).

^b^aHRs and corresponding 95% CIs were estimated using Cox proportional hazards models adjusting for age, sex, AF type and CHA_2_DS_2_-VASc score. The drug therapy group was used as the reference category for all comparisons. Interaction terms were used to assess heterogeneity of treatment effect by comorbidity burden.

^c^The primary outcome was a composite endpoint including all-cause mortality, disabling stroke, cardiac arrest or serious bleeding events.

“High” indicates ≥4 comorbidities; “Low” indicates <4 comorbidities, defined based on baseline comorbidity count in the CABANA trial.

Abbreviations: AF, atrial fibrillation; CI, confidence interval; CV, cardiovascular; aHR, adjusted hazard ratio.

Supplementary Table S7. Clinical outcomes across comorbidity burden (<4 vs. ≥4)^a^ by as-treated analysis.

|  |  | aHR (95% CI)^a^ | P Value | Interaction P Value |
| --- | --- | --- | --- | --- |
| Primary outcome^c^ | Low | 1.08 (0.70, 1.65) | 0.731 | 0.049 |
|  | High | 0.59 (0.38, 0.90) | 0.015 |  |
|  |  |  |  |  |
| Components of primary outcome | |  |  |  |
| All-Cause mortality | Low | 1.00 (0.59, 1.70) | 0.995 | 0.285 |
|  | High | 0.65 (0.39, 1.08) | 0.100 |  |
|  |  |  |  |  |
| Disabling stroke | Low | 0.45 (0.09, 2.34) | 0.346 | - |
|  | High | - | - |  |
|  |  |  |  |  |
| Cardiac arrest | Low | 0.76 (0.18, 3.21) | 0.711 | 0.415 |
|  | High | 0.32 (0.07, 1.50) | 0.147 |  |
|  |  |  |  |  |
| Serious bleeding | Low | 2.04 (0.99, 4.20) | 0.053 | 0.007 |
|  | High | 0.49 (0.24, 1.00) | 0.051 |  |
|  |  |  |  |  |
| Secondary outcome |  |  |  |  |
| CV hospitalisation | Low | 0.96 (0.82, 1.12) | 0.601 | 0.697 |
|  | High | 0.91 (0.75, 1.11) | 0.363 |  |
|  |  |  |  |  |
| All-cause mortality/CV hospitalisation | Low | 0.93 (0.80, 1.09) | 0.366 | 0.598 |
|  | High | 0.87 (0.72, 1.05) | 0.152 |  |

^a^aHRs and corresponding 95% CIs were estimated using Cox proportional hazards models, with catheter ablation included as a time-dependent covariate. Patients who underwent catheter ablation after the occurrence of the outcome were considered untreated for that specific outcome. Models were adjusted for age, sex, AF type and CHA₂DS₂-VASc score. Interaction terms were used to assess heterogeneity of treatment effect by comorbidity burden.

^b^The primary outcome was a composite endpoint including all-cause mortality, disabling stroke, cardiac arrest or serious bleeding events.

“High” indicates ≥4 comorbidities; “Low” indicates <4 comorbidities, defined based on baseline comorbidity count in the CABANA trial.

Abbreviations: AF, atrial fibrillation; CI, confidence interval; CV, cardiovascular; aHR, adjusted hazard ratio.

Supplementary Table S8. Recurrence atrial fibrillation across comorbidities burden by ITT

|  | Total number | Catheter Ablation | | Drug Therapy | | aHR (95% CI)^a^ | P Value | Interaction P Value |
| --- | --- | --- | --- | --- | --- | --- | --- | --- |
|  |  | N, n (%) | Events, n (%) | N, n (%) | Events, n (%) |  |  |  |
| Low | 813 | 410 (50.4) | 194 (47.3) | 403 (49.6) | 284 (70.5) | 0.51 (0.42–0.61) | < 0.001 | 0.163 |
| High | 538 | 278 (51.7) | 158 (56.8) | 260 (48.3) | 181 (69.6) | 0.60 (0.48–0.74) | < 0.001 |  |

^a^aHRs and corresponding 95% CIs were estimated using Cox proportional hazards models adjusting for age, sex, AF type and CHA2DS2-VASc score. The drug therapy group was used as the reference category for all comparisons.

“High” indicates ≥4 comorbidities; “Low” indicates <4 comorbidities, defined based on baseline comorbidity count in the CABANA trial.


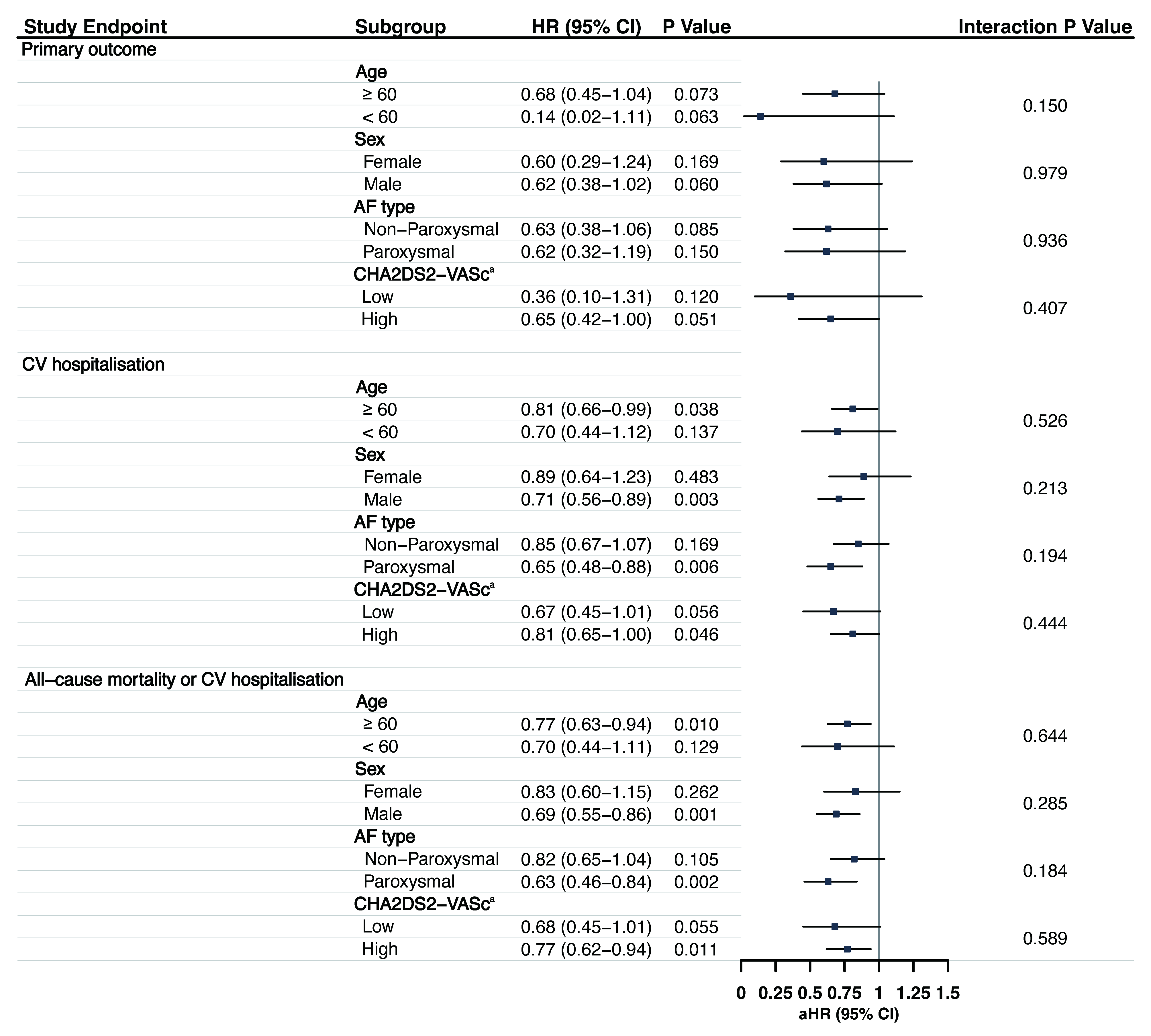


**Supplementary Figure S1. Subgroup analysis of the primary and secondary outcomes in patients with high comorbidity burden.** ^a^High CHADS₂-VASc risk was defined as a score ≥3 in males and ≥4 in females. The drug therapy group was used as the reference category for all comparisons. aHR, adjusted hazard ratio; AF, atrial fibrillation; CI, confidence interval; CV, cardiovascular.
